# Biochemical characterization and synergism of cellulolytic enzyme system from *Chaetomium globosum* on rice straw saccharification

**DOI:** 10.1186/s12896-016-0312-7

**Published:** 2016-11-21

**Authors:** Wanwitoo Wanmolee, Warasirin Sornlake, Nakul Rattanaphan, Surisa Suwannarangsee, Navadol Laosiripojana, Verawat Champreda

**Affiliations:** 1 The Joint Graduate School for Energy and Environment (JGSEE), King Mongkut’s University of Technology Thonburi, Prachauthit Road, Bangmod, Bangkok, 10140 Thailand; 2 Enzyme Technology Laboratory, National Center for Genetic Engineering and Biotechnology (BIOTEC), 113 Thailand Science Park, Phahonyothin Road, Khlong Luang, Pathumthani 12120 Thailand; 3 Bioprocess Laboratory, National Center for Genetic Engineering and Biotechnology (BIOTEC), 113 Thailand Science Park, Phahonyothin Road, Khlong Luang, Pathumthani 12120 Thailand; 4 BIOTEC-JGSEE Integrative Biorefinery Laboratory, Innovation Cluster 2 Building, 113 Thailand Science Park, Phahonyothin Road, Khlong Luang, Pathumthani 12120 Thailand

**Keywords:** Cellulase, *Chaetomium globosum*, Lignocellulose, Saccharification, Synergistic action

## Abstract

**Background:**

Efficient hydrolysis of lignocellulosic materials to sugars for conversion to biofuels and chemicals is a key step in biorefinery. Designing an active saccharifying enzyme system with synergy among their components is considered a promising approach.

**Results:**

In this study, a lignocellulose-degrading enzyme system of *Chaetomium globosum* BCC5776 (CG-Cel) was characterized for its activity and proteomic profiles, and synergism with accessory enzymes. The highest cellulase productivity of 0.40 FPU/mL was found for CG-Cel under the optimized submerged fermentation conditions on 1% (w/v) EPFB (empty palm fruit bunch), 2% microcrystalline cellulose (Avicel®) and 1% soybean meal (SBM) at 30 °C, pH 5.8 for 6 d. CG-Cel worked optimally at 50–60 °C in an acidic pH range. Proteomics analysis by LC/MS/MS revealed a complex enzyme system composed of core cellulases and accessory hydrolytic/non-hydrolytic enzymes attacking plant biopolymers. A synergistic enzyme system comprising the CG-Cel, a β-glucosidase (Novozyme® 188) and a hemicellulase Accellerase® XY was optimized on saccharification of alkaline-pretreated rice straw by a mixture design approach. Applying a full cubic model, the optimal ratio of ternary enzyme mixture containing CG-Cel: Novozyme® 188: Accellerase® XY of 44.4:20.6:35.0 showed synergistic enhancement on reducing sugar yield with a glucose releasing efficiency of 256.4 mg/FPU, equivalent to a 2.9 times compared with that from CG-Cel alone.

**Conclusions:**

The work showed an approach for developing an active synergistic enzyme system based on the newly characterized *C. globosum* for lignocellulose saccharification and modification in bio-industries.

**Electronic supplementary material:**

The online version of this article (doi:10.1186/s12896-016-0312-7) contains supplementary material, which is available to authorized users.

## Background

The shift from petroleum-based industry to a greener bio-based platform is expedited by an increasing concern of global warming. Lignocellulosic plant biomass has attracted attention as a renewable resource for production of biofuels and commodity chemicals in biorefinery. Lignocellulosic materials consist mainly of three different types of biopolymers: (i) cellulose, a linear homopolymer of D-glucose organized into highly crystalline microfibers which are intimately associated with an intricate network of (ii) hemicellulose, an amorphous branched polymer comprising various pentoses, hexoses, and sugar acids, and (iii) lignin, a heteropolymer of phenolic alcohols which shields the polysaccharide microstructure from external physical, chemical, and biological attacks. These biopolymers are organized into a complex and highly recalcitrant lignocellulosic structure [1].

In nature, lignocelluloses are degraded by the cooperation of various microorganisms, capable of producing an array of cellulolytic, hemicellulolytic, and ligninoloytic enzymes [2, 3]. Cellulases and hemicellulases are mainly composed of various hydrolytic enzymes in different glycosyl hydrolase (GH) families acting synergistically. Cellulases comprise three major groups of enzymes: (1) endoglucanases (EC 3.2.1.4), which attack regions of low crystallinity in cellulose fibers, creating free chain-ends; (2) exo-glucanases or cellobiohydrolases (EC 3.2.1.91) which further degrade the molecule by cleaving cellobiose from the free-chain ends; and (3) β-glucosidases (EC 3.2.1.21) which hydrolyze cellobiose to produce glucose [4]. In addition to the three major groups of cellulases, there are a number of endo- and exo-acting enzymes that attack the heterogeneous hemicelluloses, such as endo-β-1,4-xylanase, β-xylosidase, galactomannanase, glucomannanase, and acetylesterase. A challenge for development of a feasible biomass industry is identifying efficient lignocellulolytic microbes and developing an active enzyme system based on synergism of the major glycosyl hydrolases and the non-hydrolytic auxiliary components (e.g. expansins and lytic polysaccharide monooxygenases) [5, 6]. These molecular systems are required for efficient hydrolysis of lignocelluloses to sugars, the key intermediates for subsequent conversion to biorefinery products.

Aerobic fungi belonging to phylum Ascomycota are important plant biomass degraders capable of secreting an array of glycosyl hydrolases and non-hydrolytic auxiliary components. Several ascomycetes are commonly used for producing cellulases and hemicellulases in industry, e.g. *Trichoderma*, *Aspergillus*, and *Talaromyces* [7]. Enzymes from these fungi are different in their composite activities and enzyme components, which have different catalytic characteristics on lignocellulose decomposition. *Chaetomium* is a saprophytic fungus belonging to Ascomycota with high capability on degrading plant materials [8]. Most studies on *Chaetomium*, particularly on *C. globosum* have focused on laccases [9–11] while there have been few studies on their cellulolytic enzyme systems [12]. In this study, a biomass degrading enzyme system of the soft-rot fungus *C. globosum* BCC5776 was studied. The crude enzyme was characterized for its catalytic activities and its components identified by proteomics. The crude enzyme was used for formulation of an efficient biomass-degrading enzyme mixture by a mixture design approach and applied for hydrolysis of alkaline-pretreated rice straw. The work provides an alternative lignocellulose-degrading enzyme system potent for on-site enzyme production for the development of a feasible biorefinery industry.

## Methods

### Strain and media

Rice straw was obtained from a paddy field in Pathumthani province, Thailand according to the required national guideline. It was physically processed by a cutting mill (Retsch SM 200, Hann, Germany) and then sieved to particles smaller than 0.5 mm. The biomass was delignified with 10% (w/v) NaOH at 80 °C for 90 min at the solid/liquid ratio of 1:3, washed with water until neutral pH was obtained, and dried at 60 °C for overnight before use as the substrate for enzymatic hydrolysis. The alkaline-pretreated rice straw contained 74.3% cellulose, 13.4% hemicellulose, 1.36% lignin and 2.9% ash according to the standard NREL analysis method [13]. Empty palm fruit bunch (EPFB) was obtained from Kasetsart University, Thailand and physically processed by a chopping machine (Model JL800, Zhengzhou, China) and subsequently on a cutting mill and sieved through a 0.5 mm mesh. *C. globosum* BCC5776 was identified and obtained from the BIOTEC Culture Collection, Thailand (www.tbrcnetwork.org) and maintained on potato dextrose agar (PDA). Polysaccharides used as substrates in enzymatic activity analysis were obtained from Sigma-Aldrich. Accellerase®XY (hemicellulase from *Trichoderma reesei*) and Novozyme®188 (β-glucosidase from *Aspergillus niger*) were obtained from Dupont (Rochester, NY) and Sigma-Aldrich, respectively.

### Optimization of cellulase production conditions

*C. globosum* BCC5776 was cultivated by submerged fermentation in 250-mL conical flasks. The inoculum was prepared from the culture grown on PDA by plunging four agar pieces covered with profuse mycelia using a cock borer no. 2 and inoculated into 50 mL of the production medium (4% (w/v) of microcrystalline cellulose Avicel® and 1% (w/v) of soybean meal in water). The culture was incubated at 30 °C for 6 d with shaking at 200 rpm. Culture media and conditions were varied as specified including concentration of Avicel® (2, 4, and 6% (w/v)), concentration of lactose (0, 0.05, and 0.1% (v/v)) and pH (5.8 and 7.0 using 50 mM potassium phosphate buffer). Effect on addition of 1% (w/v) empty palm fruit bunch (EPFB) as a co-carbon source for induction of cellulase was also studied. The cultures were collected periodically for determination of cellulase activity using the dinitrosalicylic acid (DNS) method [14]. The experiments were performed in triplicate and the average of cellulase activity was used as the response (dependent variable). The data were analyzed using SPSS 16.0 (StatSoft, Inc., Tulsa, OK).

### Enzyme production and purification

A 10% (v/v) inoculum grown in the optimal production medium containing 2% Avicel®, 1% EPFB, 1% soybean meal (SBM), 0.1% lactose, and initial pH of 5.8 at 30 °C for 6 d was inoculated into a 5 L bioreactor (BIOSTAT B-DCU, Sartorius, Göttingen, Germany) with a 3 L working volume of the same medium. The culture was incubated at 30 °C for 6 d with constant mixing at 200 rpm and oxygen feed of 1 vvm. The fungal mycelia were separated by filtration on gauze and clarified by centrifugation at 10,000 × g for 10 min. The supernatant was then filtered through a 0.2 μm Supor®-200 membrane (Pall Corp, Ann Arbor, MI) followed by concentration (5×) using a Minimate™ tangential flow filtration (TFF) system equipped with a 10-kDa MWCO TFF membrane (Pall Corp, Ann Arbor, MI, USA). The enzyme was kept at 4 °C and used in subsequent experiments.

### Enzyme activity assays

Polysaccharide-degrading enzyme activities were analyzed using the 3,5-dinitrosalicylic acid (DNS) method by measuring the amount of reducing sugars liberated [14] according to the standard procedure recommended by the Commission on Biotechnology, IUPAC [15] with modifications on the total reaction volume. Reactions of 3.5 mL contained 100 mM sodium acetate phosphate buffers, pH 5.5 with an appropriate dilution of the enzyme using a 1 × 6 cm Whatman no. 1 filter paper as the substrate and incubated at 50 °C for 60 min for determining the Filter paper activity (FPase) as filter paper unit (FPU). The carboxymethyl cellulase (CMCase), xylanase, mannanase, amylase, and pectinase activities were assayed using 1% (w/v) carboxymethyl cellulose, 1% (w/v) birchwood xylan, 0.5% (w/v) locust bean gum, 1% (w/v) soluble starch, and 0.5% (w/v) pectin from citrus peels as the substrates, respectively. The reactions were incubated at 50 °C for 30 min. The amount of reducing sugars was determined at the end of the reaction by measuring the absorbance at 540 nm using a UV-Vis spectrophotometer microplate reader (Multiskan Ascent, Thermo Scientific, Cambridge, MA) and interpolation from a standard curve prepared using dilutions of the corresponding sugar as standards. One enzyme activity unit (U) is defined as the amount of enzyme required to release 1 μmol of reducing sugars from a substrate in 1 min under the assay condition. The β-glucosidase and β-xylosidase activities were assayed using 0.1% (w/v) *p*-nitrophenyl-β-D-glucopyranoside (PNPG) and *p*-nitrophenyl-β-D-xylopyranoside (PNPX) as the substrates, respectively in 3 mL reactions containing an appropriate amount of enzyme in 100 mM sodium acetate buffer (pH 5.5). The reactions were incubated at 50 °C for 30 min and terminated by the addition of 2 mL of 1 M Na_2_CO_3_. The quantity of *p*-nitrophenolate was measured spectrophotometrically at 405 nm at the end of the reaction. One activity unit (U) is defined as amount of enzyme that produces 1 μmol *p*-nitrophenolate per minute under the experimental conditions. The total protein concentration of the crude enzyme extracts was determined using Bradford’s method with the BioRad’s Protein Assay reagent (BioRad, Hercules, CA) using bovine serum albumin (BSA) as the standard. The experiments were performed in triplicate.

### Proteomic analysis

The enzyme preparation was applied to a 10% SDS-PAGE gel and separated using a MiniProtean II cell (Biorad, Hercules, CA, USA). The protein bands were visualized by staining with Coomassie blue R-250 and were manually excised into five fractions according to their apparent molecular weights (14.4–116.0 kDa). The polypeptides in gel were then digested with trypsin (Ettan Spot Handling Workstation User Manual 18-1153-55 Edition AC, GE Healthcare Biosciences, Uppsala, Sweden). The tryptic peptides were resuspended with 0.1% formic acid and analyzed on a Finigan LTQ linear ion trap mass spectrometer (Thermo Scientific, San Jose, CA, USA) according to a method described in Wongwilaiwalin et al., [16]. All MS/MS spectra were searched using the Mascot® search engine (Matrix Science, Boston, MA) against the NCBI-nr database following criteria: enzyme trypsin, static modification of Cys (+57.05130 Da), with differential modification of Met (+15.99940). The search results were filtered by cross-correlation versus charge state (+1 ≥ 1.5, +2 ≥ 2.0,+3 ≥ 2.5) and protein probability (minimum 1.00E^−3^). The candidate protein queries were mapped to the UniProt Knowledge base [17].

### Synergistic action of *C. globosum* enzyme to commercial enzymes

Interactions among the *C. globosum* enzyme (CG-Cel), Novozyme®188 and Accellerase®XY were studied using experimental mixture design approach [18] with a fixed total enzyme volume [19]. The enzymatic hydrolysis reactions of 1 mL total volume contained 5% (w/v) alkaline-pretreated biomass in 100 mM sodium acetate buffer (pH 5.5) supplemented with 1 mM sodium azide and incubated at 50 °C for 48 h with rotary shaking at 200 rpm. An optimal enzyme mixture releasing the highest reducing sugar yield was defined by a {3,3}-augmented simplex lattice design using Minitab 16.0 software (Minitab Inc., State College, PA). The design contained 13 experimental points (see design summarized in Table 3), which were performed in quadruplicate with three components and a lattice degree of three. The three independent variables in the mixture design consisted of CG-Cel (X1), Novozyme®188 (*X*2), and Accellerase®XY (X3). The sum of all enzyme components in the reactions were 100% with the total enzyme volume fixed at 80 μL. The amount of released reducing sugar (Y1) was used as dependent variables for simulation of the respondent model equation. The amount of reducing sugars liberated at the end of the reactions was determined using the DNS method at the end of the reaction [14]. The sugar profile in the hydrolyzates was analyzed by high performance liquid chromatography (Waters e2695, Waters, Milford, MA) equipped with a differential refractometer using an Aminex HPX-87H column (Bio-Rad, Hercules, CA). The column temperature was 65 °C. H_2_SO_4_ solution (5 mM) was used as the mobile phase at a flow rate of 0.5 mL/min. Concentration of sugar was determined from the calibration curve of standard solution. The experiments were performed in quadruplicate. Sugars from control reactions containing heat-inactivated enzymes were subtracted from the data.

### Mixture design analysis

After regression analysis, the full cubic model was used to simulate the optimized ratio of the mixture components. The canonical correlation of the full cubic model is shown in Eq. (1):

1 $$ Y={\displaystyle {\sum}_{i=1}^3{\beta}_i{X}_i+{\displaystyle \sum {\displaystyle {\sum}_{i<j}^3{\beta}_{ij}{X}_i{X}_j+{\displaystyle \sum {\displaystyle {\sum}_{i<j}^3{\delta}_{ij}{X}_i{X}_j\left({X}_i-{X}_j\right)+{\displaystyle \sum {\displaystyle \sum {\displaystyle {\sum}_{i<j<k}^3{\beta}_{ijk}{X}_i{X}_j{X}_k}}}}}}}} $$

where Y is a predicted response, β_i_ is a linear coefficient, β_ij_ is a quadratic coefficient, and β_ijk_ is a cubic coefficient. β_ij_ is a parameter of the model. β_i_X_i_ represents the linear blending portion, and the parameter β_ij_ represents either synergistic or antagonistic blending.

## Results and discussion

### Optimization of enzyme production conditions

Production of cellulolytic enzyme from *C. globosum* BCC5776 by submerged fermentation was studied. The optimization study included three variables: (1) concentration of Avicel®, (2) concentration of lactose, and (3) initial pH previously identified to show significant effects (*p* < 0.05) on cellulase level of the fungus in the preliminary screening of factors influencing enzyme production. According to Table 1, the basic fermentation condition containing Avicel® as the sole carbon source (run no. 1) resulted in the CMCase and FPase activity of 6.37 and 0.21 U/mL, respectively. Further increases in Avicel® (run no. 6–9) did not enhance the target enzymatic activities. Addition of lactose (run no. 2–9) was found to significantly (*p* < 0.05) induce cellulase production level when compared with the same conditions in the absence of lactose. Higher cellulase productivity was found at acidic pH (5.8) compared with that observed under neutral conditions. The highest CMCase and FPase activities of 7.19 U/mL and 0.32 FPU/mL, respectively, were recorded after 144 h of fermentation in run no. 3 containing 1% lactose and an inducer with controlled pH at 5.8. This up-regulation of cellulase by lactose was previously reported in *C. papyrosolvens* [20] and *A. cellulolyticus* [21] where lactose was shown to act as an effective inducer of cellulase biosynthesis, which is controlled by a regulator protein responsive to the concentration of the target substrate [22]. The preference for acidic pH for production of cellulase and other lignocellulose degrading enzymes has been reported for most cellulase producing ascomycetes fungi e.g. *T. reesei* [23], *A. cellulolyticus* [24], *and C. globosum* [25]. This preference is related to pH for optimal growth and catalytic activity of the enzymes.

**Table 1:** Effects of variables to cellulase activities in fermentation of BCC5776

No.	Concentration (%)		Initial pH	Activity	
	Avicel®	Lactose		CMCase (U/mL)	FPase (FPU/mL)
1	4	-	5.5	6.37 ± 0.01	0.21 ± 0.03
2	2	0.05	5.8	7.02 ± 0.01	0.22 ± 0.05
3	2	0.1	5.8	7.19 ± 0.00	0.32 ± 0.01
4	2	0.05	7.0	5.10 ± 0.03	0.20 ± 0.00
5	2	0.1	7.0	5.54 ± 0.02	0.17 ± 0.01
6	6	0.05	5.8	6.87 ± 0.04	0.18 ± 0.00
7	6	0.1	5.8	6.68 ± 0.01	0.20 ± 0.01
8	6	0.05	7.0	6.12 ± 0.05	0.17 ± 0.00
9	6	0.1	7.0	5.07 ± 0.04	0.17 ± 0.03

**Fig. 1:**
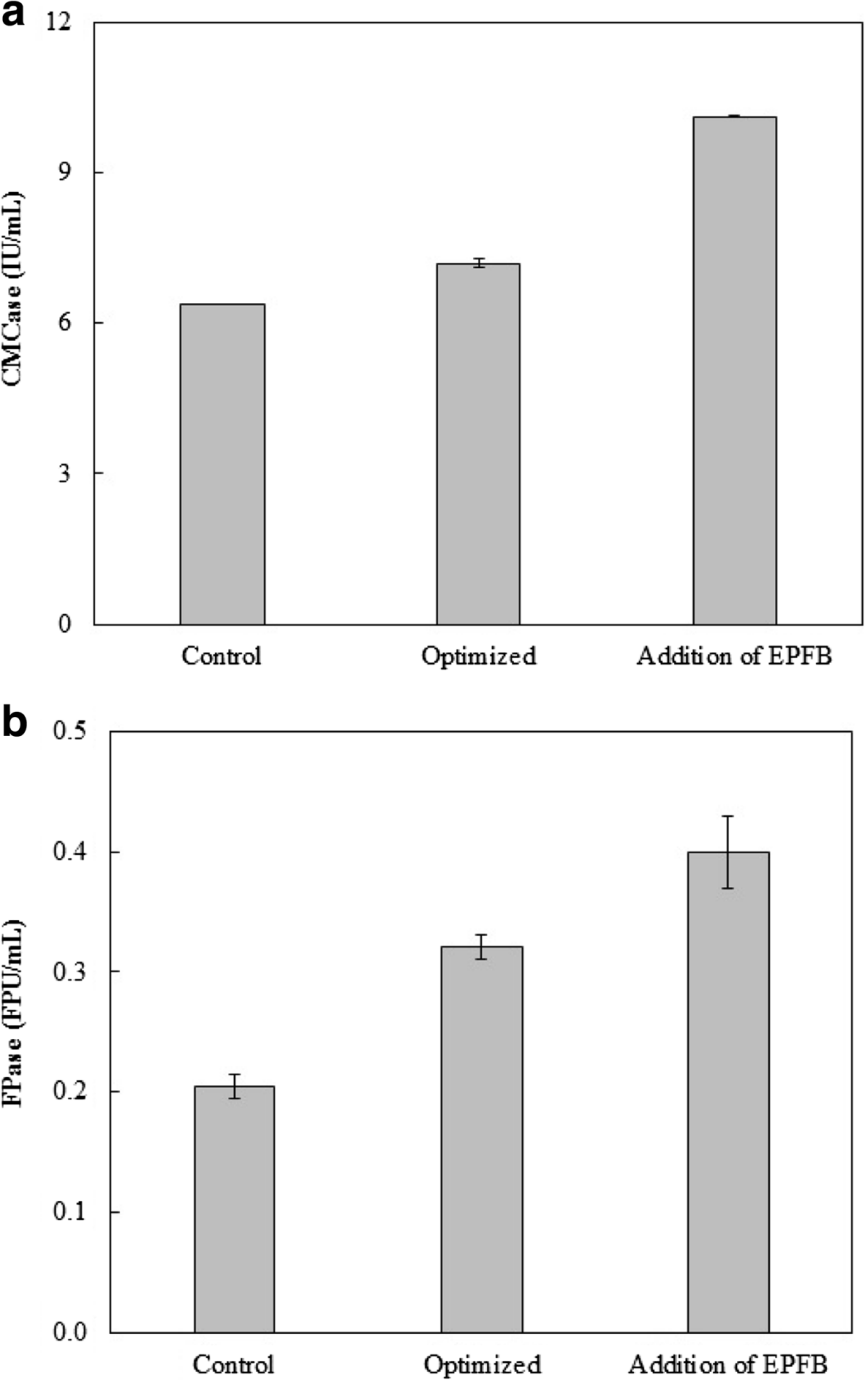
Comparison of (**a**) CMCase and (**b**) FPase activities from *C. globosum* BCC5776 under various conditions. The cultures were incubated at 30 °C for 144 h with continuous shaking at 200 rpm. Control medium (4% (*w*/*v*) of microcrystalline cellulose Avicel® and 1% (*w*/*v*) of soybean meal in water); Optimized medium (2% (*w*/*v*) microcrystalline cellulose Avicel®, 0.1% (*v*/*v*) lactose, and 1% (*w*/*v*) soybean meal with 50 mM potassium phosphate, pH 5.8); Optimized medium + 1% (*w*/*v*) EPFB

Addition of EPFB as a co-substrate at 1% (w/v) concentration under submerged fermentation was found to further improve the cellulase production by *C. globosum* BCC5776. This led to the enhancement of endo-glucanase (CMCase) and total cellulase (FPase) activities to 10.57 U/mL and 0.40 FPU/mL, equivalent to 47 and 25% increases, respectively, compared with those obtained in its absence (Fig. 1a and b). The use of agro-industrial wastes as the sole carbon source or in combination with pure cellulose as an effective and cost-efficient substrate for production of plant biomass degrading enzymes e.g. cellulase, hemicellulase, and pectinase production has been demonstrated in many fungal strains [26–28]. The cellulase activities produced by *C. globosum* in this study were in the same range (or higher in some cases, particularly for the CMCase activity) compared to several wild type fungi in genera *Aspergillus*, *Acremonium, Trichodema*, and *Schizophyllum* reported in many recent publications, which were in the range of 0.01–1.33 FPU/mL. The very high cellulase activities (up to five FPU/ml) were achieved by mutants or genetically modified fungal strains. Comparison of cellulase activities produced by different fungal strains recently reported is shown in the Additional file 1: Table S1.

The optimized enzyme production medium (2% Avicel®, 1% EPFB, 0.1% lactose, and 1% soybean meal at pH 5.8) was used for production of the crude enzyme (CG-Cel) in a laboratory-scale fermenter. The enzyme showed similar composite activities to those obtained in the small scale experiments. The crude enzyme preparation (Table 2) contained a relatively high level of endo-glucanase as shown by CMCase activity (15.7 U/mL) with FPase activity (0.4 FPU/mL). Strong endo-β-1,4-xylanase (28.2 U/mL) and endo-β-1,4-mannanase (13.6 U/mL) activities were found together with other enzymes involved in plant polysaccharide degradation, such as pectinase (0.8 U/mL) and amylase (0.5 U/mL). However, regardless of the high activities of endo-acting glycosyl hydrolases, the crude enzyme exhibited substantially weak downstream activities i.e. β-glucosidase (1.5 U/mL) and β-xylosidase (0.04 U/mL) activities, which could limit degradation of cellulose and hemicelluloses to sugars. The 5× concentrated enzyme contained the cellulase activity of 2.0 FPU/mL while >90% activities of other enzymes were retained.

**Table 2:** The composite enzyme activity profiles of *C. globosum* BCC5776 enzyme extracts

Enzyme	Activity (U/mL)^a^
CMCase	15.70
FPase	0.40
β-glucosidase	1.50
Xylanase	28.20
β-xylosidase	0.04
Amylase	0.50
Mannanase	13.60
Pectinase	0.80

^a^Protein concentration = 1.30 mg/mL

### Synergistic action of CG-Cel and commercial enzymes

**Fig. 2:**
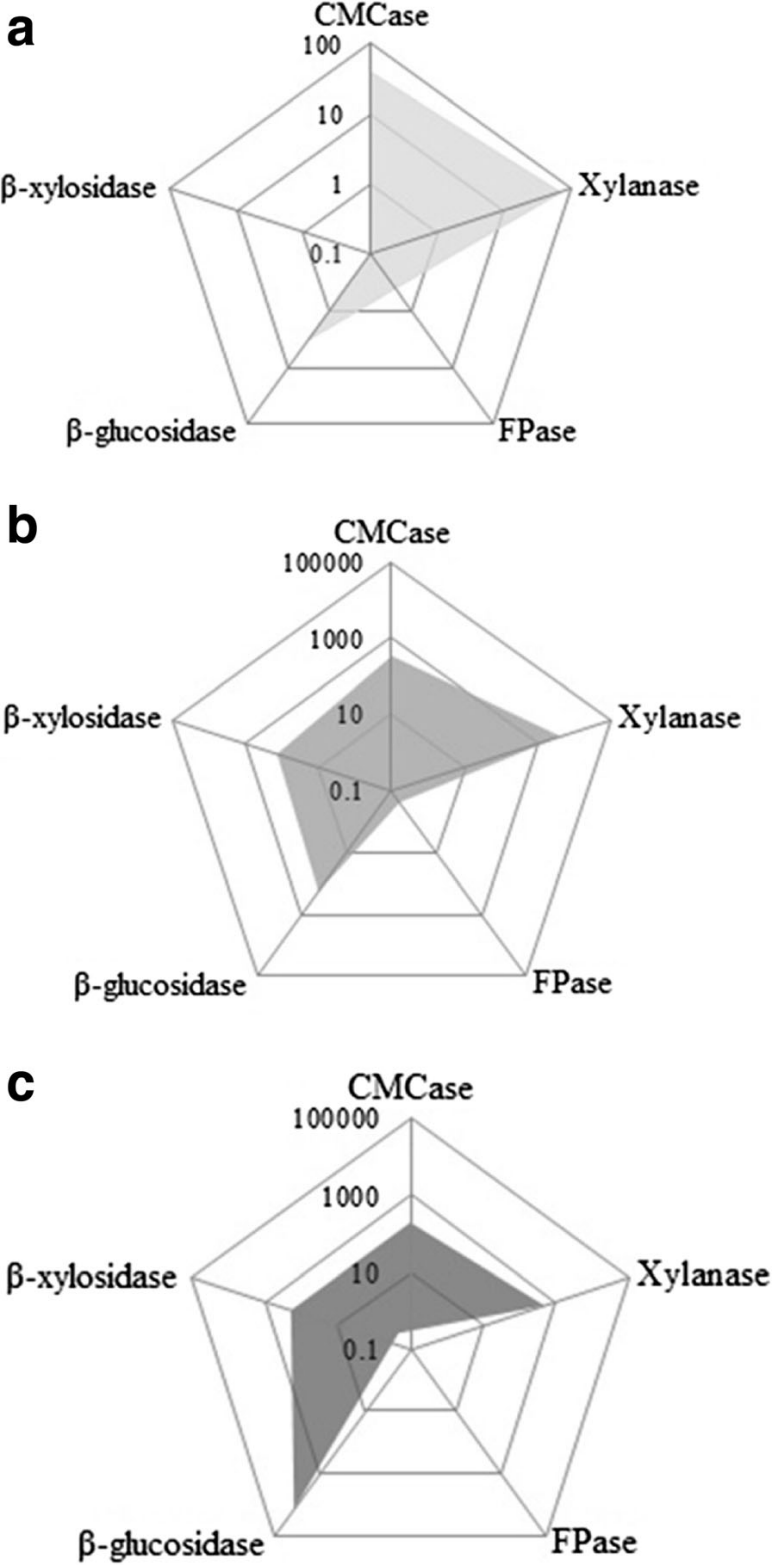
Comparative activity profiles of (**a**) CG-Cel and the synergized enzymes (**b**) Accellerase®XY and (**c**) Novozyme®188. The Y-axis is in a logarithm base 10 scale (log U/FPU)

The mixture design approach was applied to study synergistic and cooperative interactions among CG-Cel and a hemicellulase, Accellerase®XY, and a β-glucosidase, Novozyme®188, chosen from our pre-screening study on identification of additive enzymes with complementing activities to the core cellulase (see the comparative enzyme profiles in Fig. 2). The measured response of this method was presumed to depend only on the enzyme proportion of the complements in the mixture. A {3,3}-simplex lattice model was applied which comprised 13 experimental points located inside the triangular graph, in which the sum of the three component loading for every experimental point was always 100% based on a volumetric basis. According to this design, the cellulase dosages as FPU loading/g substrate of each experimental point was varied according to different proportions of CG-Cel: Novozyme®188:Accellerase®XY. Each point was operated in quadruplicate to minimize the effect of experimental error.

The reducing sugar yield obtained from each experimental condition in the mixture design is summarized in Table 3. The standard deviation of all the data points was ≤ 5% of the mean. CG-Cel, equivalent to the FPase activity of 3.2 FPU/g (No. 1), demonstrated a higher reducing sugar yield (500.9 mg/g) than those obtained with the β-glucosidase Novozyme®188 (No. 7) and the hemicellulase Accellerase®XY (No. 10) alone. The binary combinations between CG-Cel and Accellerase®XY (No. 3 and 6) led to slight increases in reducing sugar yields (519.3–525.4 mg/g) compared with that of CG-Cel alone, despite the lower total FPase dosage in the reaction (1.17–2.18 FPU/g). Ternary mixtures of all three enzymes at specific compositions resulted in further increases in the total reducing sugar yields, with the maximal reducing sugar yield of 567.8 mg/g (No. 11) which contained a 4:1:1 ratio of BCC5776: Novozyme®188: Accellerase®XY, suggesting synergism of the three enzymes.

**Table 3:** Design of experiment for enzymatic hydrolysis of pretreated rice straw and the associated response data

Run no.	% composition			Reducing sugar	
				(mg/g biomass)	
	BCC5776	Novozyme®188	Accellerase®XY	Average	SD
1	100.0	0.0	0.0	500.9	2.6
2	66.7	33.3	0.0	493.1	2.2
3	66.7	0.0	33.3	525.4	0.5
4	33.3	66.7	0.0	421.2	4.3
5	33.3	33.3	33.3	535.6	2.6
6	33.3	0.0	66.7	519.3	3.2
7	0.0	100.0	0.0	69.2	3.4
8	0.0	66.7	33.3	205.3	4.1
9	0.0	33.3	66.7	233.3	4.6
10	0.0	0.0	100.0	205.0	4.9
11	66.7	16.7	16.7	567.8	1.7
12	16.7	66.7	16.7	426.1	1.4
13	16.7	16.7	66.7	483.5	2.1

Reactions (1 mL) contained 5% (w/v) alkaline-pretreated rice straw in 100 mM sodium acetate buffer, pH 5.5 with a total enzyme volume of 80 μL and incubated at 50 °C for 48 h

The response data for the reducing sugar were then analyzed using multiple regression analysis from the linear to full cubic model. The full cubic model was found to be the best fitted model for the reducing sugar (R^2^ = 97.30%, P_Model_ < 0.01). The ANOVA analysis of the full cubic model is illustrated in Table 4. For a single factor, CG-Cel, Novozyme®188, Accellerase®XY coefficients showed a positive correlation with the reducing sugar yield. Marked synergisms between CG-Cel and Novozyme®188 or Accellerase®XY were observed (786.6 and 845.1), with a weak synergistic interaction between Novozyme®188 and Accellerase®XY. The highest coefficient was observed for the CG-Cel*Novozyme®188*Accellerase®XY, indicating strong interactions among these components. The fitted equation for the reducing sugar yield based on the significant terms is shown in Eq. (2):

**Table 4:** The regression model analysis of the {3,3} full cubic model

Factor	Coefficient	SE	T	*p*-value
BCC5776	482.7	14.3	*	*
Novozyme188	72	14.3	*	*
AccelleraseXY	205	14.3	*	*
BCC5776*Novozyme188	786.6	63.94	12.3	0.000
BCC5776*AccelleraseXY	845.1	63.94	13.22	0.000
Novozyme188*AccelleraseXY	377.3	63.94	5.9	0.000
BCC5776*Novozyme188*Accellerase XY	2534.8	416.85	6.08	0.000
BCC5776*Novozyme188*(-)	−586.8	122.48	−4.79	0.000
BCC5776*AccelleraseXY*(-)	−528.2	122.48	−4.31	0.000
Novozyme188*AccelleraseXY*(-)	78.8	122.48	0.64	0.523
S = 28.6660	PRESS = 47389.9			
R^2^ = 97.30%	R^2^ (pred) = 96.29%		R^2^ (adj) = 96.72%	

2 $$ \begin{array}{l}\mathrm{Reducing}\ \mathrm{sugar}\ \left(\mathrm{mg}/\mathrm{g}\right)=4.82685*\mathrm{B}\mathrm{C}\mathrm{C}5776+0.72036*{\mathrm{Novozyme}}^{\circledR }188\ \\ {}+2.04999*{\mathrm{Accellerase}}^{\circledR}\mathrm{X}\mathrm{Y}+0.07866*\mathrm{B}\mathrm{C}\mathrm{C}5776*{\mathrm{Novozyme}}^{\circledR }188+0.08451*\mathrm{B}\mathrm{C}\mathrm{C}5776\\ {}*{\mathrm{Accellerase}}^{\circledR}\mathrm{X}\mathrm{Y}+0.03772*{\mathrm{Novozyme}}^{\circledR }188*{\mathrm{Accellerase}}^{\circledR}\mathrm{X}\mathrm{Y}+0.00253*\mathrm{B}\mathrm{C}\mathrm{C}5776*\ \\ {}{\mathrm{Novozyme}}^{\circledR }188*{\mathrm{Accellerase}}^{\circledR}\mathrm{X}\mathrm{Y}\left(-\right)-0.00059*\mathrm{B}\mathrm{C}\mathrm{C}5776*{\mathrm{Novozyme}}^{\circledR }188\left(-\right)-\\ {}0.00053*\mathrm{B}\mathrm{C}\mathrm{C}5776*{\mathrm{Accellerase}}^{\circledR}\mathrm{X}\mathrm{Y}\left(-\right)\end{array} $$

**Fig. 3:**
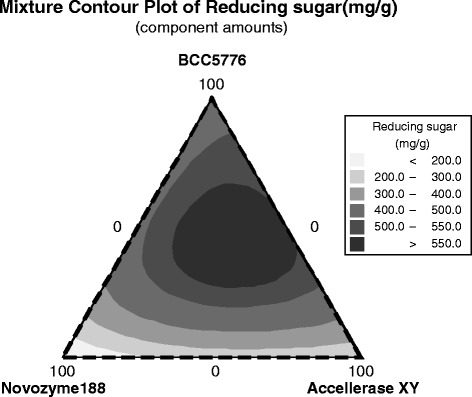
The contour plot of the experimental design optimization of the ternary enzyme complex. One hundred percent component amount is equal to 80 μL total reaction volume

**Fig. 4:**
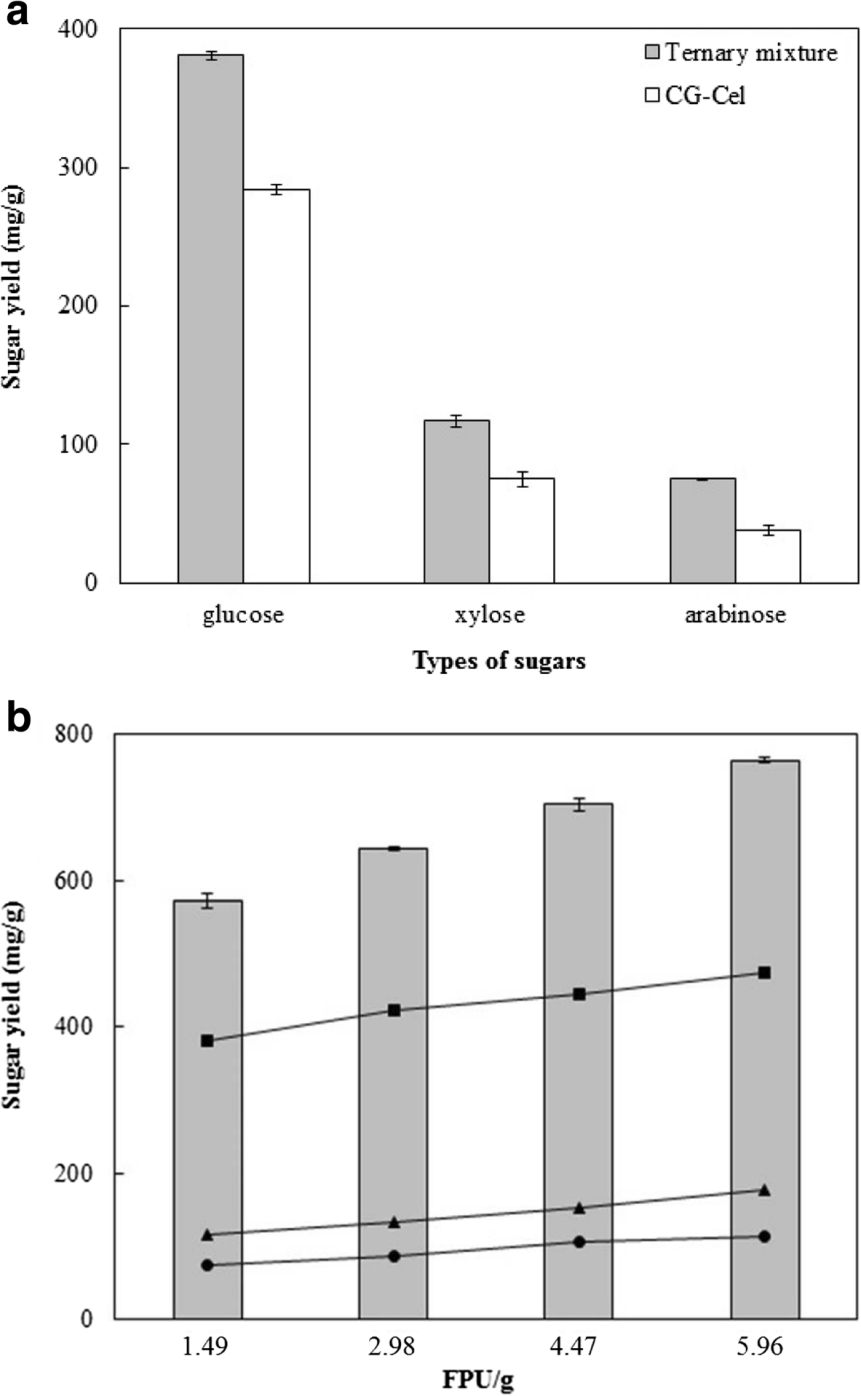
Saccharification of pretreated rice straw using the optimal enzyme mixture compared with individual enzymes. The reactions (1 mL) contained 5% (*w*/*v*) alkaline-pretreated rice straw in 100 mM sodium acetate buffer, pH 5.5 with a fixed total enzyme volume of 80 μL (1×) and incubated at 50 °C for 48 h. (**a**) sugar profile of optimal enzyme mixture; (**b**) the total reducing sugars (bar) and individual sugar profile ((■) glucose; (▲) xylose; (♦) arabinose) versus FPU loading/g on pretreated rice straw hydrolysis. *1× enzyme loading equaled to 1.49 FPU/g

The responses of reducing sugar yield with respect to component combinations are represented by a ternary mixture contour plot (Fig. 3). The area that emphasized the greatest reducing sugar was in the middle of the CG-Cel and Accellerase®XY axes, and near the bottom of the Novozyme®188 vertex. This implies that a high level of reducing sugars could be achieved when CG-Cel was the major component with Novozyme®188 and Accellerase®XY in the minority. The optimal enzyme combination based on the maximal reducing sugar yield was determined to be 44.4% CG-Cel, 20.6% Novozyme®188, and 35.0% Accellerase®XY with a predicted reducing sugar yield of 591.2 mg/g pretreated rice straw with the total cellulase activity of 1.49 FPU/g. An experimental reducing experimental sugar yield of 572.7 mg/g was obtained, validating the model. The ternary enzyme mixture was found to increase the yields of all major composite sugars (i.e. glucose, xylose, and arabinose) from hydrolysis of the pretreated rice straw. The glucose yield (Additional file 2: Table S2) obtained using the optimal enzyme mixture was 381.2 mg/g, which was higher than that obtained using the individual enzymes (Fig. 4a). This led to the increase in glucose releasing efficiency from 88.7 mg glucose/FPU from CG-Cel alone by 2.9 fold to 256.4 mg glucose/FPU of the ternary optimal enzyme mixture. (*p* < 0.05 by *T*-test) [29]. Further increases in the total enzyme loading from 1× (1.49 FPU/g) to 4× (5.96 FPU/g) led to a stepwise increase in reducing sugar yield to 764.7 mg/g and glucose released to 474.8 mg/g; however, respective decreases in glucose releasing efficiency were observed at higher enzyme loadings (Fig. 4b).

### Proteomic analysis of BCC5776 crude enzyme

In order to analyze the composite glycosyl hydrolases and accessory non-hydrolytic enzymes with functions on attacking lignocellulosic materials in CG-Cel, the composite proteins in the secretome of BCC5776 (see Additional file 3: Figure S1) was analyzed using LC/MS/MS. Twenty seven different proteins were identified in CG-Cel, from which 81% were annotated as functional proteins while the rest were classified as hypothetical proteins. The majority of them are hydrolytic enzymes attacking cellulose and hemicelluloses, with a substantial fraction of non-cellulosic polysaccharide hydrolyzing enzymes (Table 5). Most of them are closely related to homologous enzymes in *Neofusicoccum parvum* UCRNP2 and other ascomycetes*.* The cellulose degrading enzymes were classified into various glycosyl hydrolase families and non-hydrolytic enzymes in different classes. The cellulolytic components are annotated as 5 endo-glucanases (GH5 and 7) and 8 cellobiohydrolases, including CBH-I (GH6 and 7), which attacks the reducing end of cellulose chain, and CBH-II, which attacks the non-reducing end, releasing cellobiose units, in addition to one carbohydrate binding module (CBM1).

**Table 5:** Identification of the polysaccharide degrading enzymes in the secretome of *Chaetomium globosum* BCC5776

GI number	GH	Protein name	Organism	Mascot	Prot
	Family			Score	Mass
343435330	GH7	Cellobiohydrolase I	*Uncultured fungus*	111	18482
343441380	GH7	Cellobiohydrolase I	*Uncultured fungus*	60	17704
517325505	GH5	Probable cellulase precursor	*Fusariam fujikuroi*	59	42508
343435350	GH7	Cellobiohydrolase I	*Uncultured fungus*	55	18629
485920895	GH6	Putative cellobiohydrolase II protein	*Neofusicocum parvum*	167	47768
310801037	GH7	Glycoside hydrolase family 7 endoglucanase	*Collectotrichum graminicola*	150	49663
46395332	GH7	Cellobiohydrolase	*Irpex lacteus*	111	56112
407917740	GH5	Glycosyl hydrolase family 5 endoglucanase	*Macrophomina phaseolina*	95	32163
477528227	GH7	Exoglucanase type c precursor	*Collectotrichum graminicola*	109	52197
367046256	GH7	Glycosyl hydrolase family 7 endoglucanase	*Theilavia terrestris*	84	56119
49333363	GH7	Cellobiohydrolase II	*Volvariella volvacea*	82	55320
343435720	GH7	Cellobiohydrolase I	*Uncultured fungus*	75	18463
575066085	-	Carbohydrate-binding module family 1 protein	*Heterobasidion irregulare*	68	35482
485929653	-	Putative cellobiose dehydrogenase protein	*Neofusicocum parvum*	99	897431
407926573	GH35	Glycoside hydrolase family 35 β-galactosidase	*Macrophomina phaseolina*	204	107902
407929733	GH47	Glycoside hydrolase family 47 α-mannosidase	*Macrophomina phaseolina*	122	57629
74664704	GH10	Endo-1,4-β-xylanase (Xylanase) (EC 3.2.1.8)	*Aspergillus oryzae*	101	34903
		(1,4-β-D-xylan xylanohydrolase)			
407926897	GH10	Glycoside hydrolase family 10 endoxylanase	*Macrophomina phaseolina*	189	34499
485916263	GH10	Putative endo-β-xylanase protein	*Neofusicocum parvum*	185	35125
485916633	GH10	Putative extracellular endo-β-protein	*Neofusicocum parvum*	130	34638
485919833	GH11	Putative endo-β-xylanase protein	*Neofusicocum parvum*	87	23690
500259512	GH11	Putative endo-β-xylanase I protein	*Phaeoacremonium minimum*	84	23642
485916757	-	Putative carboxypeptidase s1 protein	*Neofusicocum parvum*	78	61265
485919267	-	Putative leucyl aminopeptidase protein	*Neofusicocum parvum*	147	40795
485924582	-	Putative tripeptidyl-peptidase 1 protein	*Neofusicocum parvum*	68	65131
407926489	-	Polysaccharide deacetylase	*Macrophomina phaseolina*	66	26890
325683994	GH7	Glycoside hydrolase family 7 endoglucanase	*Phialophora sp.*	161	50242

A few hemicellulolytic enzymes were also identified in the BCC5776 secretome. These included GH10 and 11 endo-β-1,4-xylanase, a key endo-acting hemicellulase attacking xylan, the major component in hemicellulose in addition to exo-acting enzymes including a β-galactosidase (GH35) and α-mannosidase (GH47). Non-hydrolytic counterparts included a cellobiose dehydrogenase acting on oxidizing cellodextrins, an intermediate in cellulose hydrolysis to their corresponding lactones, and a polysaccharide deacetylase functioning on acetyl group branches in hemicelluloses. The functions of both enzymes on synergistic and cooperative actions with core cellulases have been demonstrated [30]. Other hydrolases in the secretome are mainly related to different classes of proteases, e.g. carboxypeptidase s1, leucyl aminopeptidase, and tripeptidyl-peptidase 1. No other non-cellulosic polysaccharide degrading enzymes e.g. amylases or pectinases were identified. The proteomic profiles of the secretome thus point to a lack of downstream cellulose degrading enzyme (i.e. a β-glucosidase) and various hemicellulolytic components. The lack of these activities could explain why CG-Cel is strongly synergistic with supplemented pure β-glucosidase and hemicellulases.

Synergism of core cellulases with hemicellulases and auxiliary components in various classes of carbohydrate processing enzymes according to the Carbohydrate Active Enzymes Database (CaZY) [31] has been demonstrated and could be applied for developing efficient enzyme systems for bioindustries. Their synergistic interactions can be explained by several mechanisms [32–34]. Activities of upstream enzymes can be enhanced by cooperative action with downstream enzymes acting on degrading smaller substrates, for example, alleviation of cellobiohydrolase (CBH) inhibition by a β-D-glucosidase which cleaves cellobiose, the released product with an inhibitory effect to CBHs to glucose molecules [35]. Synergism can also be resulted from the endo/exo effect between an endoglucanase which creates new, free cellodextrin chain ends for an exo-acting CBH [36] as well as synergic action between different exoglucanases attacking the reducing and non-reducing ends of cellulose chains [37]. Cooperative action of cellulases and hemicellulases can also increases accessibility to the target substrates of each other, providing synergisms between glycosyl hydrolases attacking different biopolymers in the plant cell wall [38]. Physical modification of the substrates e.g. loosening of the crystalline region of cellulose by auxiliary proteins (e.g. expansins) [32] and non-hydrolytic enzymes (e.g. lytic polysaccharide monoxygenases) [39] which in overall, results in increased accessibility of the hydrolytic enzymes to the substrates has also been demonstrated. However, they were not annotated in the secretome of *C. globosum* in this study.

Synergistic enzyme systems are developed by empirically determining combinations of different enzymes for optimal digestibility on specific lignocellulosic substrates in order to reduce enzyme usage without sacrificing the rate or yield from substrate hydrolysis. *T. reesei* cellulase, a well-known cellulase in biomass industry was shown to lack β-glucosidase activity, resulting in strong inhibition of the cellulase by accumulated cellobiose in the reaction. This inhibition can be overcome by addition of external β-glucosidase enzymes from different microbial origins [40]. The synergistic action of *T. reesei* cellulase with a crude enzyme mixture from *A. aculeatus* containing various cell wall polysaccharide degrading enzymes with strong downstream cellulolytic and hemicellulolytic activities and a non-catalytic bacterial expansin, acting on physical loosening of the crystalline region of cellulose fibers, on saccharification of alkaline pretreated rice straw was reported [19]. Glycosyl hydrolases from the metagenome of microflora present in sugarcane bagasse can enhance reducing sugar yield of *T. reesei* cellulase [41]. A recent work also showed strong synergistic action of a commercial cellulase, Acellerase®1500 with an endo-xylanase, pectate lyase, and a hemicellulose side chain cleaving α-arabinofuranosidase [33]. These findings demonstrate the potential of formulating active synergistic enzyme systems from various microbial sources in order to maximize the hydrolysis efficiency on specific lignocellulosic substrates. The glucose releasing efficiency of the synergistic ternary enzyme system developed in this study was higher than that reported for Accellerase®1500 (81 mg glc/FPU) and the synergistic enzyme systems based on that enzyme, which was in the range of 122–173 mg/FPU [33]. The efficiency of the ternary enzyme mixture is in the same range to that reported for a synergistic system comprising *T. reesei* cellulase, a crude enzyme from *A. aculeatus* and a bacterial expansin [19] (229 mg glc/FPU) and between *T. reesei* cellulase and enzyme from *A. awamori* [42] (200 mg glc/FPU). The greater efficiency of the *C. globosum* enzyme system may depend though on the nature of the substrates and hydrolysis conditions. Elucidation of synergized enzyme activities in this study thus provides a basis for further modification of the CG-Cel enzyme either by complementation of lack activities by external in-house enzymes or genetic modification of the strain.

## Conclusions

A biomass-degrading enzyme system from *C. globosum* BCC5776 (CG-Cel), a potent fungus for development of a cellulase producing strain for on-site enzyme production, was characterized. The primary cellulase activity can be augmented by other hydrolytic and non-hydrolytic accessory enzymes. The enzyme system shows a high degree of synergism with commercial β-glucosidase and hemicellulase. The optimal combination of CG-Cel, β-glucosidase and hemicellulase was found, leading to marked improvement in the sugar releasing efficiency. The work provides an approach for designing an effective enzyme system with specificity for saccharification of lignocellulosic materials, including agricultural biomass for the biorefinery industry.

## Additional files

Additional file 1: Table S1. Comparison of cellulose production by *C. globosum* BCC5776 and other fungi in recent selected publications. (DOCX 16 kb)
Additional file 2: Table S2. Sugar yields from hydrolysis of pretreated rice straw using various enzyme combinations and dosages. (DOCX 15 kb)
Additional file 3: Figure S1. SDS-PAGE analysis of the BCC5776 crude enzyme extracts. Lane 1- Marker proteins; Lane 2 –the CG-Cel crude enzyme extracts stained with Coomassie brilliant blue. (DOCX 45 kb)

## Abbreviations

CG-Cel: Cellulase system of *Chaetomium globosum* BCC5776
CMCase: Cellulase activity on carboxymethyl cellulose
DNS: Dinitrosalicylic acid
EPFB: Empty palm fruit bunch
FPase: Cellulase activity on filter paper
GH: Glycosyl hydrolase
SBM: Soybean meal
TFF: Tangential flow filtration
