# Supplementary material for: Biochemical characterization and synergism of cellulolytic enzyme system from Chaetomium globosum on rice straw saccharification
Source: BMC Biotechnol. 2016 Nov 21;16:82. doi: 10.1186/s12896-016-0312-7 (PMC5117696; doi:10.1186/s12896-016-0312-7)
Supplement: Additional file 1: Table S1. — Comparison of cellulose production by C. globosum BCC5776 and other fungi in recent selected publications. (DOCX 16 kb) [file 12896_2016_312_MOESM1_ESM.docx]

**Table S1:** Comparison of cellulose production by *C. globosum* BCC5776 and other fungi in recent selected publications

| Strains | Enzyme activity | | Ref. |
| --- | --- | --- | --- |
|  | FPase (FPU/mL) | CMCase  (IU/mL) |  |
| *C. globosum* BCC5776 (wild-type) | 0.40 | 15.70 | this work |
| *Acremonium strictum* (wild-type) | 0.01 | 0.18 | Golbeck et al. 2013 |
| *Acremonium zeae* EA0802 (wild-type) | 0.14 | 0.09 | Almeida et al. 2011 |
| *Aspergillus sydowii* (wild-type) | 1.33 | 1.32 | Matkar et al. 2013 |
| *Schziophyllum commune* (wild-type) | 0.08 | NA^a^ | Bergadi et al. 2014 |
| *T. reesei* Rut-C30 (recombinant) | 5.00 | 64.00 | Prévot et al. 2013 |
| *P. janthinellum* NCIM 1171 (mutant) | 1.20 | 37.20 | Singhvi et al. 2011 |

^a^NA = not analyzed.

Golbeck R, Ramos MM, Pereira GAG, Maugeri-Filho F. Cellulase production from a new strain *Acremonium strictum* isolated from the Brazilian Biome using different substrates. Bioresour Technol. 2013;128:797-803.

Almeida MND, Guimarães VM, Bischoff KM, Falkoski DL, Pereira OL, Gonçalves DSPO, Rezende STD. Cellulases and hemicellulases from endophytic *Acremonium* species and its application on sugarcane bagasse hydrolysis. Appl Biochem Biotechnol. 2011;165:594-610.

Matkar K, Chapla D, Divecha J, Nighojkar A, Madamwar D. Production of cellulase by a newly isolated strain of Aspergillus sydowii and its optimization under submerged fermentation. Inter Biodeterior Biodegradation. 2013;78:24-33.

Bergadi FE, Laachari F, Elabed S, Mohammed IH, Ibnsouda SK. Cellulolytic potential and filter paper activity of fungi isolated from ancients manuscripts from the Medina of Fez. Ann Microbiol. 2014;64:815-822.

Prévot V, Lopez M, Copinet E, Duchiron F. Comparative performance of commercial and laboratory enzymatic complexes from submerged or solid-state fermentation in lignocellulosic biomass hydrolysis. Bioresour Technol. 2013;129:690–693.

Singhvi MS, Adsul MG, Gokhale DV. Comparative production of cellulases by mutants of *Penicillium janthinellum* NCIM 1171 and its application in hydrolysis of Avicel and cellulose. Bioresour Technol. 2011;102:6569–6572.
