# Supplementary material for: Biochemical characterization and synergism of cellulolytic enzyme system from Chaetomium globosum on rice straw saccharification
Source: BMC Biotechnol. 2016 Nov 21;16:82. doi: 10.1186/s12896-016-0312-7 (PMC5117696; doi:10.1186/s12896-016-0312-7)
Supplement: Additional file 2: Table S2. — Sugar yields from hydrolysis of pretreated rice straw using various enzyme combinations and dosages. (DOCX 15 kb) [file 12896_2016_312_MOESM2_ESM.docx]

**Table S2**: Sugar yields from hydrolysis of pretreated rice straw using various enzyme combinations and dosages.

| **No.** | **Enzyme components** | **Total reducing sugar** | **Glucose** | **Xylose** | **Arabinose** |
| --- | --- | --- | --- | --- | --- |
|  |  | **(mg/g )** | **(mg/g )** | **(mg/g )** | **(mg/g )** |
| 1 | CG-Cel | 396.9 | 283.7 | 74.9 | 38.3 |
| 2 | Novo 188 | 49.1 | 19.8 | 16.6 | 12.7 |
| 3 | Acc XY | 173.6 | 20.5 | 68.4 | 74.4 |
| 4 | BCC+Novo 188+Acc XY* (1x) | 572.7 | 381.2 | 116.6 | 74.9 |
| 5 | BCC+Novo 188+Acc XY (2x) | 642.6 | 423.3 | 134.1 | 85.2 |
| 6 | BCC+Novo 188+Acc XY (3x) | 703.6 | 445.2 | 152.0 | 106.5 |
| 7 | BCC+Novo 188+Acc XY (4x) | 764.7 | 474.8 | 177.7 | 112.1 |

Reactions (1 mL) contained 5% (w/v) pretreated rice straw in 100 mM sodium acetate buffer (pH 5.5) with different enzyme combinations (with the total sum of enzyme equaled to 80 μL) and incubated at 50^º^C for 48 h.

CG-Cel, *C. globosum* BCC5776 (100% = 3.20 FPU/g substrate); Novo 188, Novozyme^®^188 (100% = 0.05 FPU/g substrate); Acc XY, Accellerase^®^XY (100% = 0.16 FPU/g substrate).

^*^Ternary mixture at the optimal ratio (44.4:20.6:35.0). 1x enzyme represented 1.49 FPU/g substrate.
