# Supplementary material for: Biochemical characterization and synergism of cellulolytic enzyme system from Chaetomium globosum on rice straw saccharification
Source: BMC Biotechnol. 2016 Nov 21;16:82. doi: 10.1186/s12896-016-0312-7 (PMC5117696; doi:10.1186/s12896-016-0312-7)
Supplement: Additional file 3: Figure S1. — SDS-PAGE analysis of the BCC5776 crude enzyme extracts. Lane 1- Marker proteins; Lane 2 –the CG-Cel crude enzyme extracts stained with Coomassie brilliant blue. (DOCX 45 kb) [file 12896_2016_312_MOESM3_ESM.docx]

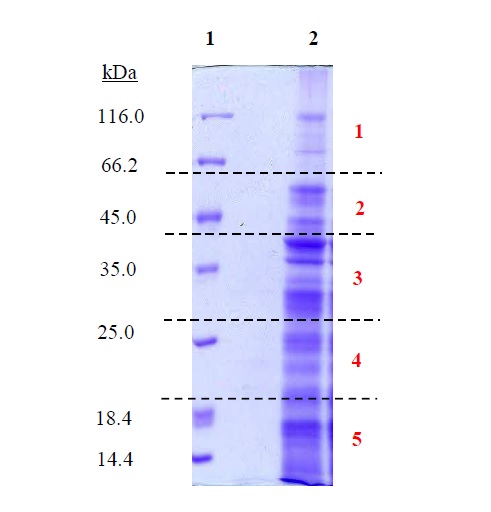


**Figure S1.** SDS-PAGE analysis of the BCC5776 crude enzyme extracts. Lane 1- Marker proteins; Lane 2 –the CG-Cel crude enzyme extracts stained with Coomassie brilliant blue.
